# Multilocus sequencing-based evolutionary analysis of 52 strains of *Burkholderia pseudomallei* in Hainan, China

**DOI:** 10.1017/S0950268818002741

**Published:** 2018-10-08

**Authors:** Y. Fang, Z. Hu, H. Chen, J. Gu, H. Hu, L. Qu, X. Mao

**Affiliations:** 1 Department of Clinical Microbiology and Immunology of College of Medical Laboratory Science, Third Military Medical University, Chongqing, 400038, China; 2 Department of Respiratory, NO.161 Hospital of PLA, Wuhan, 430012, China; 3 Department of Respiratory, Wuhan General Hospital of PLA, Wuhan, 430070, China; 4 Department of Clinical Laboratory, People's Hospital of Sanya City, Hainan Province 572000, China; 5 National Engineering Research Centre for Immunobiological Products, Third Military Medical University, Chongqing, 400038, China

**Keywords:** *Burkholderia pseudomallei*, e-BURST, multilocus sequence typing (MLST)

## Abstract

Previously, we reported a phylogenetic study of 98 *Burkholderia pseudomallei* clinical isolates from Hainan, China. Here, we update the *B. pseudomallei* strain library with 52 strains from newly identified cases dating from 2014 to 2017, analysed by multilocus sequence typing. Twenty-two sequence types (STs) were identified from the 52 cases, illustrating high genetic diversity; five of them (ST1480, ST1481, ST1482, ST1483 and ST1484) were novel. ST46, ST50 and ST58 predominated (34.6%) as was the case in the previous study (35.7%). An e-BURST map of the ST profiles of the two collections of isolates showed their genetic foundation to be largely unchanged. Neighbour-joining tree analysis was suggestive of a close phylogenetic relationship between the novel STs from this series and those first reported from Hainan (ST1105, ST1099, ST55 and ST1095). Moreover, the two novel STs (1481 and 1483) showed close similarity to ST58 which originated in Thailand indicating a close relationship between *B. pseudomallei* strains from both countries. The previously described allele profiles *gmhD*-36 and *lepA*-68 were found for the first time in our strain collections. Our study emphasises the importance of monitoring the epidemiological status and evolutionary trends of *B. pseudomallei* in China.

*Burkholderia pseudomallei* is a select agent (Tier 1) as classified by the US Centers for Disease Control and Prevention (CDC), which can infect both animals and humans and causes the disease melioidosis [1]. Melioidosis was generally thought to be endemic in Southeast Asia and Northern Australia but is now known to be distributed on a much wider scale [2–5]. Clinical manifestations of melioidosis vary greatly, from pneumonia to sepsis, with a high mortality rate of 40%, and a clinical relapse rate up to 20% [6–8]. Hainan is the main melioidosis-endemic area of China and covers an area of 33 210 km^2^ with a population of over 9 million residents [8–10].

Multilocus sequence typing (MLST) has been used for the molecular epidemiological study of *B. pseudomallei* since 2003, and MLST data have proved to be easy-to-use, unambiguous and readily comparable across laboratories [11]. To date, approximately 1453 sequence types (STs) of *B. pseudomallei* have been identified worldwide, and the number of new cases identified by MLST increases each year. We have adopted this methodology as a standard analysis strategy for all *B. pseudomallei* strains isolated from cases of melioidosis in Hainan and previously published a phylogenetic and epidemiological study on 98 such isolates (2002–2014) [11]. Here, we report further phylogenetic analysis of 52 strains from cases in the province typed by MLST in the last 3 years to examine their genetic stability over time.

Fifty-two *B. pseudomallei* strains were isolated from melioidosis cases between January 2014 and August 2017 in Hainan Island; two were first isolated from non-coastal cities (Baisha and Qiongzhong). Most isolates were recovered from blood (63.5%) and pus (34.6%), others were from sputum (3.8%) and urine samples (3.8%) (Table 1). Clinical samples were cultured on Columbia blood agar incubated at 37 °C for 2–3 days. *B. pseudomallei* identification was confirmed by the Vitek 2 Compact system (BioMerieux, Missouri, USA), and 16S rRNA PCR as previously described [11].

**Table 1.** Properties of the 52 strains of *B. pseudomallei* strains studied

Strain	Year	ST^a^	Allele at the following locus							Region of China^b^	Source	Manifestation
			*ace*	*gltB*	*gmhD*	*lepA*	*lipA*	*narK*	*ndh*			
BPC102	2014	1104	3	1	36	3	5	2	3	Haikou	Blood	Pneumonia
BPC103	2014	658	1	4	2	1	1	4	3	Haikou	Blood	Septicaemia
BPC104	2014	58	3	1	5	1	1	4	1	Sanya	Sputum	Septicaemia
BPC105	2014	1096	3	4	28	1	1	3	1	Changjiang	Pus	Pneumonia
BPC106	2014	46	3	1	2	1	1	3	3	Haikou	Blood	Pneumonia
BPC107	2014	58	3	1	5	1	1	4	1	Danzhou	Urine	Pneumonia
BPC108	2014	1105	1	4	2	3	5	4	1	Baisha	Pus	Pneumonia
BPC109	2014	177	1	1	4	3	1	3	1	Changjiang	Blood	Sepsis
BPC110	2014	1094	1	2	28	3	1	9	3	Danzhou	Blood	Parotid infection
BPC111	2014	58	3	1	5	1	1	4	1	Ledong	Blood	Sepsis
BPC112	2014	354	1	1	3	2	1	4	1	Haikou	Blood	Pneumonia
BPC113	2014	**1480**	3	3	2	1	1	3	3	Dongfang	Blood	Sepsis
BPC114	2015	1099	1	12	3	2	1	2	1	Haikou	Blood	Pneumonia
BPC115	2015	1095	1	12	3	2	1	2	3	Haikou	Pus	Pneumonia
BPC116	2015	1106	1	2	28	3	8	4	3	Wenchang	Pus	Sepsis
BPC137	2015	50	3	1	2	1	1	4	3	Dongfang	Pus	Pneumonia
BPC138	2015	58	3	1	5	1	1	4	1	Sanya	Pus	Pneumonia
BPC139	2015	658	1	4	2	1	1	4	3	Sanya	Blood	Pneumonia
BPC140	2015	1108	3	1	2	68	1	4	3	Sanya	Blood	Pneumonia
BPC141	2015	58	3	1	5	1	1	4	1	Sanya	Sputum	Sepsis
BPC142	2015	70	3	4	11	3	5	4	6	Sanya	Blood	Soft tissue infection
BPC143	2015	50	3	1	2	1	1	4	3	Sanya	Pus	Soft tissue infection
BPC144	2015	562	1	1	4	1	1	29	1	Sanya	Pus	Pneumonia
BPC145	2015	**1481**	3	1	5	1	5	4	1	Qionghai	Pus	Pneumonia
BPC156	2016	**1482**	3	4	2	3	5	4	6	Sanya	Blood	Pneumonia
BPC169	2016	**1483**	1	1	11	1	5	4	1	Qionghai	Blood	Pneumonia
BPC147	2016	50	3	1	2	1	1	4	3	Sanya	Blood	Pneumonia
BPC148	2016	50	3	1	2	1	1	4	3	Sanya	Blood	Sepsis
BPC149	2016	50	3	1	2	1	1	4	3	Qionghai	Blood	Pneumonia
BPC150	2016	366	3	1	2	3	8	4	3	Sanya	Pus	Pneumonia
BPC151	2016	306	1	2	3	1	1	2	1	Sanya	Pus	Pneumonia
BPC152	2016	1091	3	4	2	1	1	4	3	Sanya	Blood	Pneumonia
BPC153	2016	46	3	1	2	1	1	3	3	Sanya	Blood	Pneumonia
BPC154	2016	46	3	1	2	1	1	3	3	Ledong	Blood	Pneumonia
BPC155	2016	366	3	1	2	3	8	4	3	Sanya	Blood	Pneumonia
BPC157	2016	930	1	1	3	2	5	1	1	Sanya	Blood	Pneumonia
BPC158	2016	46	3	1	2	1	1	3	3	Ledong	Blood	Pulmonary abscess
BPC159	2016	930	1	1	3	2	5	1	1	Sanya	Blood	Pneumonia
BPC160	2016	1109	1	2	2	1	1	4	1	Qiongzhong	Blood	Pneumonia
BPC161	2016	658	1	4	2	1	1	4	3	Sanya	Blood	Pneumonia
BPC162	2016	658	1	4	2	1	1	4	3	Sanya	Pus	Pneumonia
BPC163	2016	50	3	1	2	1	1	4	3	Sanya	Pus	Pneumonia
BPC164	2016	50	3	1	2	1	1	4	3	Sanya	Blood	Pneumonia
BPC165	2016	211	3	1	3	1	1	4	1	Sanya	Blood	Pneumonia
BPC166	2016	1110	1	12	3	2	1	22	3	Ledong	Pus	Pneumonia
BPC167	2016	58	3	1	5	1	1	4	1	Sanya	Blood	Pneumonia
BPC168	2016	1111	3	4	11	4	1	4	1	Baoting	Blood	Soft tissue infection
BPC170	2017	1091	3	4	2	1	1	4	3	Ledong	Pus	Sepsis
BPC171	2017	55	3	1	3	3	1	4	1	Ledong	Blood	Pneumonia
BPC172	2017	46	3	1	2	1	1	3	3	Lingshui	Pus	Pneumonia
BPC173	2017	48	3	1	2	1	1	4	1	Lingshui	Pus	Neck abscess
BPC174	2017	**1484**	1	1	2	3	1	4	3	Lingshui	Blood	Sepsis

a The numbers in bold represent the novel STs, the death cases are labelled with underscore.

b The shaded regions represent the regions which were identified as isolation locations in Hainan Island.

For MLST, PCR amplification of seven housekeeping genes was performed as previously published [11]. The sequence data for each allele were trimmed to a determined length and defined as relative allele numbers according to the *B. pseudomallei* MLST database (https://pubmlst.org/bpseudomallei). STs were assigned and all strain numbers were deposited in the database.

Clinical characteristics and patient demographic data were analysed in Microsoft Excel 2016. The phylogenetic relationships of all strains were generated using e-BURST v3 and compared against all strains in the database with JAVA 8.0. The STs of 98 strains previously described from Hainan [11] were also analysed to show the relationship and mutational trend between the two groups of strains. The interactive tree of life (iTOL) v3 (https://pubmlst.org/bigsdb?db=pubmlst_bpseudomallei_isolates) was used to display phylogenetic relationships. This tool generates neighbour-joining trees from concatenated nucleotide sequences based on the pair-wise differences in the allelic profiles of strains [12].

The distribution of melioidosis cases across the 17 city areas of Hainan Island between the two surveys 2002–2014 (black) and 2014–2017 (red) is shown in Supplementary Fig. S1. The ages among the 52 more recent cases ranged from 1 to 79 years (median – 47 years); the majority were male (76.9%) and farmers (71.2%). Pneumonia (71.2%) and sepsis (13.5%) remained the major manifestations of melioidosis, with some cases presenting with abscesses (5.8%) and soft tissue infections (7.7%). Seven deaths occurred following pneumonia or sepsis, and old age (⩾58 years); two of the deaths were associated with novel STs (Table 1).

All 52 strains were resolved into 22 STs, five of which (ST1480, ST1481, ST1482, ST1483 and ST1484) were novel (Table 1). The dominant STs occurring in ⩾4 cases were ST50 (seven cases; 13.5%), ST58 (six; 11.5%), ST46 (five; 9.6%) and ST658 (four; 7.7%); these four STs accounted for 42.3% of all cases and the remainder were associated with ⩽3 cases. This distribution reflects that found in the first survey where the dominant STs were ST46 (13 cases; 13.2%), ST50 (11; 11.2%) and ST58 (11; 11.2%), accounting for 35.7% in all 98 strains of *B. pseudomallei*.

The pair-wise differences in the allelic profiles of the STs identified in both strain collections are shown in Fig. 1a and b with novel STs highlighted by red circles. The three dominant ST48, ST50 and ST46 were grouped into a single clonal complex (CC), and two novel ST1480 and ST1481 fell in the same CC (Fig. 1a) with other novel STs in individual CCs. ST1482 was phylogenetically related to ST70 which originated from Thailand. Figure 1b shows the evolutionary trends and phylogenetic relationships between STs identified in the two studies (from 2002 to 2017). Novel STs identified in each study period are highlighted in green (2002–2014) and red (2014–2017), respectively. Half of the cases were clustered into a single dominant CC, with ST50 as founder, and ST46 as sub-founder. Likewise three of the novel STs in the present study (ST1480, ST1481 and ST1484) and five novel STs from the first study (ST1345, ST1346, ST1347, ST1350 and ST1351) were grouped in the same CC (Fig. 1b). Furthermore, some novel STs originated from the same founders but generated different gene types: ST1484, ST1345 and ST1350 (founder: ST51), ST1480 and ST1346 (founder: ST46), ST1481 and ST1351 (founder: ST211) (Fig. 1b). Additionally, ST1482 and ST1483 were grouped in different CCs; ST1482 showed close linkage to both ST70 and ST541, both of which were first isolated from Thailand and ST1483 had a relatively close relationship with ST1349, an ST related to ST562, which was shared by Australian and Chinese strains of *B. pseudomallei* [10].

**Fig. 1.**
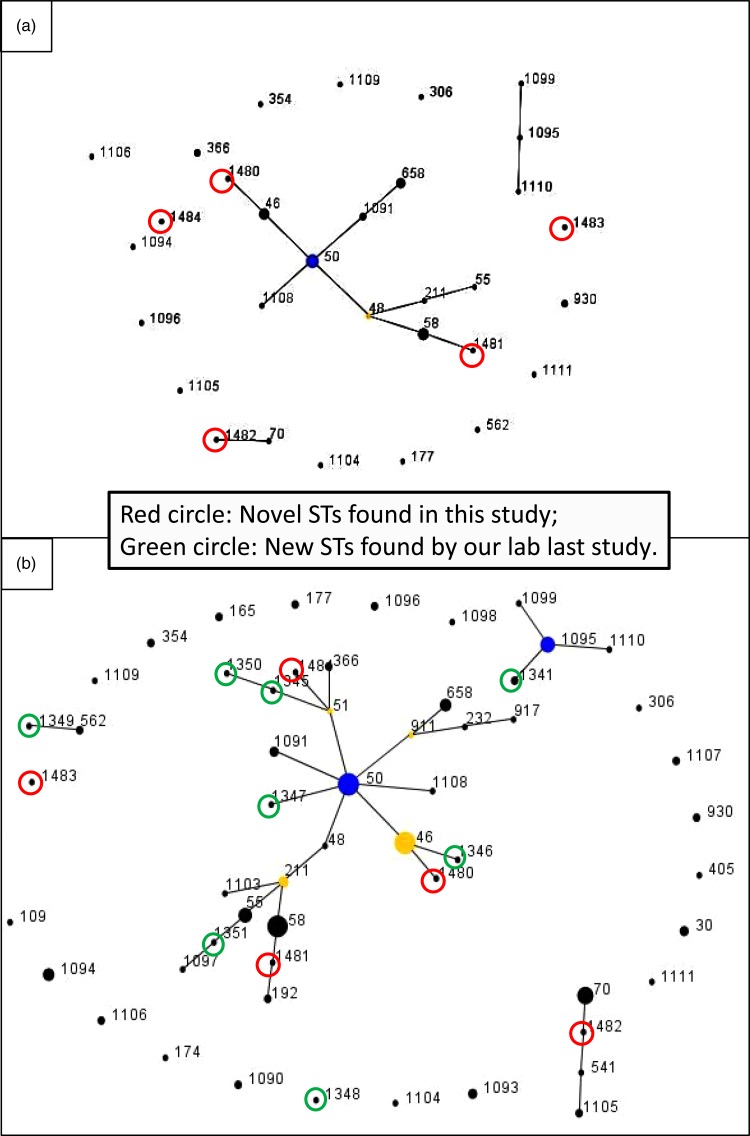
e-BURST analysis of the *B. pseudomallei* strains. (a) MLST data of 52 STs in this study. The primary founder (yellow), ST48, is positioned centrally in the cluster and subgroup founder (blue) is ST50. Strain groups are connected by dark lines and the five novel STs are highlighted by red circles. Dot diameter reflects the number of cases. (b) e-BURST of sequence types for combined 150 strains from both surveys. Novel STs highlighted by red circles and green circles, respectively. Dot diameter reflects the number of cases.

The neighbour-joining tree shows the phylogenetic relationship among the 22 STs identified in strains from this study (Fig. 2). The five novel STs were genetically close to ST1105, ST1099, ST55 and ST58, and ST1096 (all first isolated from China, except for ST58, which was first isolated from Thailand). The dominant allele profiles were mostly the same as previously found, except for *ndh*-3; allele *ndh*-1 predominated in the first strain collection. Some rare alleles were evident between the studies, such as *gmhD*-36 (ST1196) and *lepA*-68 (ST1108) (Supplementary Table S1).

**Fig. 2.**
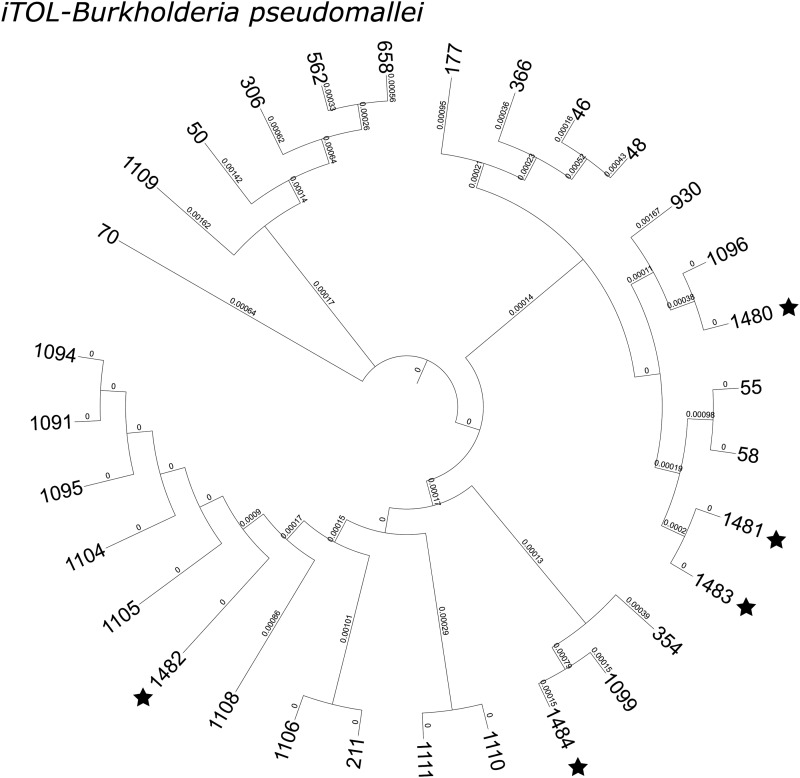
Interactive tree of life (iTOL) of MLST data from the present study. Neighbour-joining trees from concatenated nucleotide sequences based on the pair-wise differences in the allelic profiles. Twenty-two STs (identified from 52 strains, five novel STs labelled with asterisks).

It is clear that *B. pseudomallei* is no longer restricted to tropical regions but is increasingly found in non-endemic areas [6]. Melioidosis is relatively uncommon in China but most reported human cases in the country are from the tropical Hainan island [8–10], and as in other countries, pneumonia remains the most common presentation of the disease [7].

MLST has been repeatedly shown to be a simple and shareable strategy for molecular epidemiological studies of *B. pseudomallei* in various regions [2, 11]. Previously, we published an MLST study of 98 *B. pseudomallei* strains from Hainan, and here we have characterised 52 recently isolated strains and identified five novel STs. An e-BURST map of the 150 strains from both collections showed a clonal cluster with ST50 (first identified in China) as the founder genotype and that the genetic structure had remained largely unchanged over the 16-year period. Neighbour-joining tree analysis also suggests that recently emergent novel STs are closely related to some of the historical STs first isolated from Hainan Island. The linkage of two of the novel STs (1481 and 1483) to ST58, originating from Thailand, which has a close interplay with China [13]. Hainan attracts millions of tourists per year, which likely increases the risk of spreading infection among wider populations. A few melioidosis cases related to a history of travel in Hainan have been recorded by local hospitals in recent years (unpublished observations). In addition, the communications and commerce activities between the countries may promote the interaction of *B. pseudomallei* with different genetic backgrounds, and possibly give rise to the evolution of new gene types [13, 14]. No correlation was found between STs and geographic location, isolation source and clinical outcome of the patients but further genomic analysis may reveal hitherto unrecognised relationships [15].

In conclusion, this study has confirmed the relative genetic stability of *B. pseudomallei* strains in Hainan over a 15-year period. With the exception of the appearance of some novel STs, the clonal populations have remained mostly conserved but further monitoring of population structures and evolutionary trends through cooperative research with other endemic areas in the region is warranted.

## Supplementary material

For supplementary material accompanying this paper visit https://doi.org/10.1017/S0950268818002741. click here to view supplementary material
