# Supplementary material for: Multilocus sequencing-based evolutionary analysis of 52 strains of Burkholderia pseudomallei in Hainan, China
Source: Epidemiol Infect. 2018 Oct 8;147:e22. doi: 10.1017/S0950268818002741 (PMC6518616; doi:10.1017/S0950268818002741)
Supplement: Supplementary file 1 [file S0950268818002741sup001.docx]

**
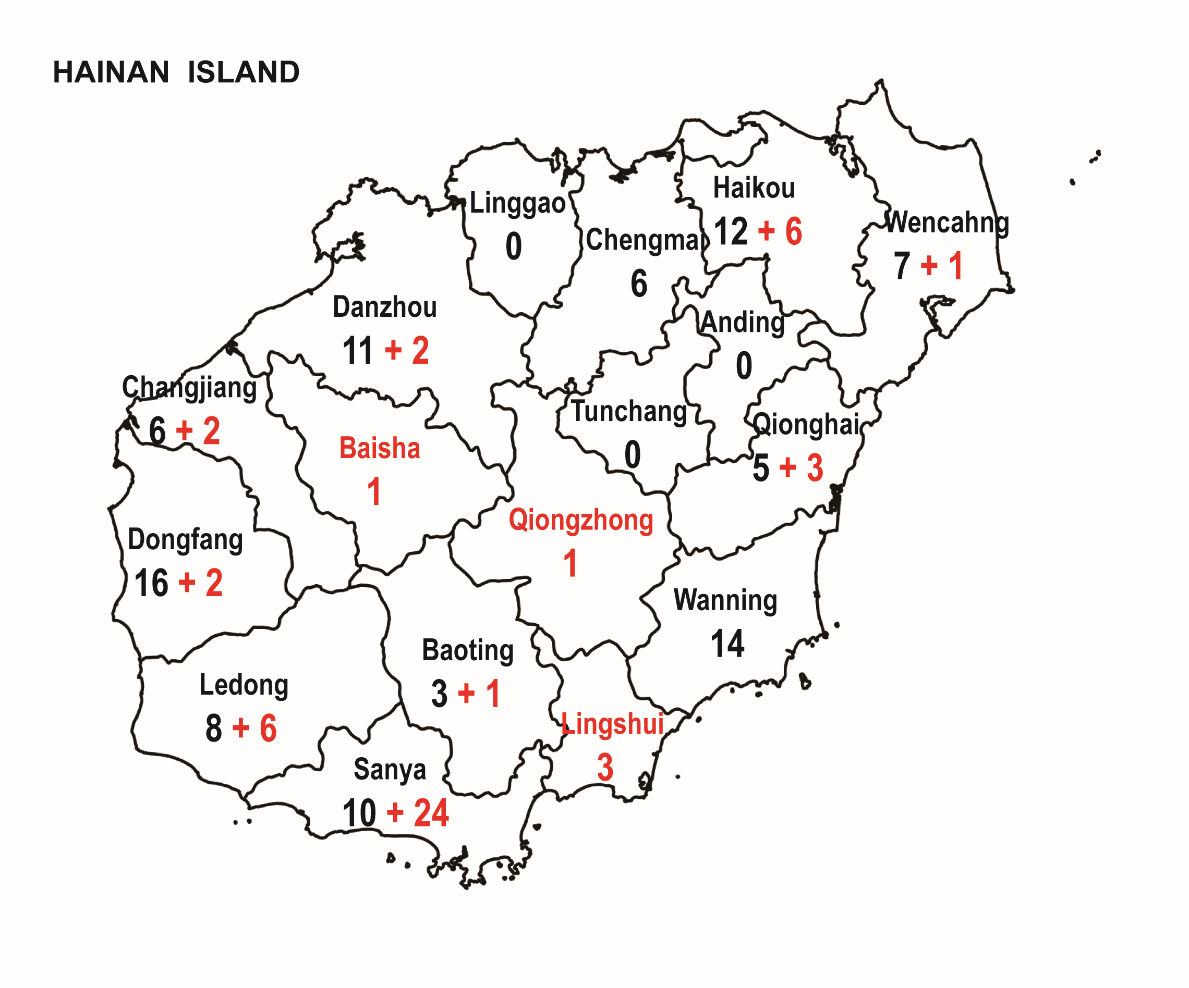
**

**Figure S1 Geographic distribution of 52 strains of B. pseudomallei isolated from Hainan, China**

Map of the Hainan Island. *B. pseudomallei* strains (n=98) from previous study (2002 to 2014) shown in black. Strains (n=52) from present study (2014-2017) shown in red.

**Table S1.** Prevalence of allele numbers of *B. pseudomallei* strains in this study

| **Locus** | **Allele number and prevalence (%)*** |
| --- | --- |
| ***ace*** | 3 (63.46) 1 (36.5) |
| ***gltB*** | 1 (63.5) 4 (21.2) 2 (7.7) 12 (5.8) |
| ***gmhD*** | 2 (51.9) 3 (17.3) 4 (3.8) 5 (13.5) 11 (5.8) 28 (5.8) **36** (1.9) |
| ***lepA*** | 1 (63.5) 3 (21.2) 2 (11.5) 4 (1.9) **68** (1.9) |
| ***lipA*** | 1 (78.8) 5 (15.4) 8 (5.8) |
| ***narK*** | 4 (67.3) 2 (7.7) 3 (15.4) 1 (3.8) 9 (1.9) 22 (1.9) 29 (1.9) |
| ***ndh*** | 3 (53.8) 1 (42.3) 6 (3.8) |

* The bold allele numbers were newly found compared to our previous study.
